# Primary pulmonary blastoma of monophasic variety- diagnosis and management

**DOI:** 10.1186/1749-8090-8-144

**Published:** 2013-06-07

**Authors:** Jitendra H Mistry, Sumita B Pawar, Harshad Mehta, Aron Frederik Popov, Prashant Nanasaheb Mohite

**Affiliations:** 1 Department of Cardiothoracic Surgery, Medical College, Vadodara, Gujarat, India; 2 Department of Pathology, ACPM Medical College, Dhule, India; 3 University of Göttingen, Göttingen, Germany; 4 Department of Cardiothoracic Transplantation & Mechanical Support, Royal Brompton and Harefield NHS Trust, Harefield Hospital, Harefield, Middlesex UB9 6JH, UK; 5 Georg-August-University Goettingen, Robert-Koch-Str. 40, D - 37075, Goettingen, Germany

**Keywords:** Pulmonary blastoma, Lung cancer

## Abstract

Pulmonary blastoma is a rare primary lung neoplasm, in that monophasic variety is far too rare. There are no specific clinical features seen for pulmonary blastoma; computed tomography and histopathology are diagnostic. Surgical excision is the treatment of choice; however, adjuvant chemotherapy and radiotherapy may be required in large and aggressive tumors.

## Background

Pulmonary blastomas are relatively rare group of primary lung neoplasms with disputed histogenesis and variable biologic behavior that are composed of immature malignant epithelial and/or mesenchymal tissues whose features may resemble early embryological lung tissues. Since the first report by Barrett & Barnard, in 1945, many more cases of pulmonary blastoma have been reported [1]. Although over a hundred cases of pulmonary blastoma are reported in the literature, the monophasic variety is extremely rare [1,2]. Pulmonary blastoma is observed in the fourth decade of life with mean age of occurrence in adults being 43 years, and shows a strong female preponderance [2]. We present a case of a teenager affected with monophasic variety of pulmonary blastoma.

## Case presentation

**Figure 1:**
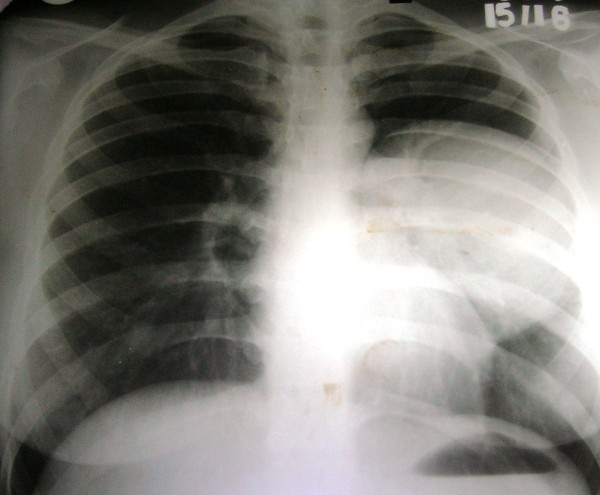
**X**- **ray chest showing soft tissue opacity in left upper lobe.**

**Figure 2:**
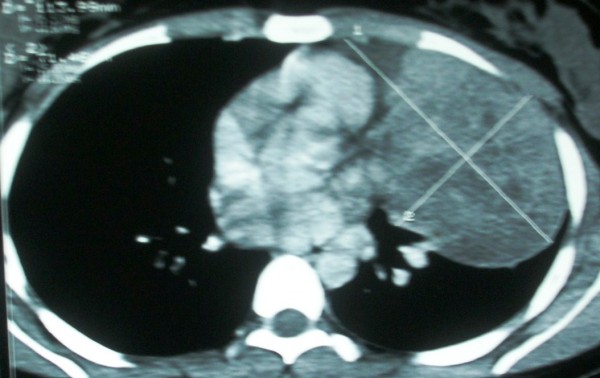
CT chest showing tumor in left upper lobe.

**Figure 3:**
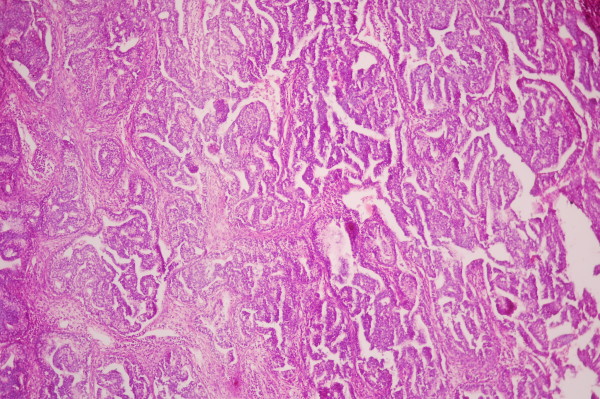
Histopathological picture.

A young healthy female of 18 years presented with left sided chest pain not related to exertion since one month. Examination of respiratory system revealed decreased air entry on left side over mammary and supra-mammary regions. Chest X-ray showed about 10 cm sized rounded opacity on left upper quadrant (See Figure 1). Spiral computed tomography (CT) scan of chest confirmed the large, neoplastic mass with few necrotic and calcific foci involving left upper lobe (See Figure 2). Ultrasonography of the abdomen and pelvis showed no abnormality. CT guided biopsy was done and the smear showed the formation of tubules lined by columnar cells that contained PAS positive clear to pale eosinophilic cytoplasm with intervening area containing spindle cells suggestive of pulmonary blastoma (See Figure 3). The samples of biopsy were also sent for immunohistochemistry which showed the lining epithelial cells of tubules strongly expressing cytokeratin and the spindle cells expressing vimetin. The focal expression of desmin was also noticed.

**Figure 4:**
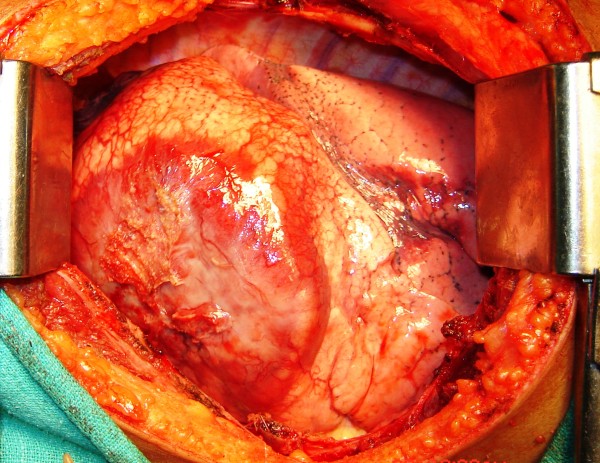
Left thoracotomy showing tumor in upper lobe.

**Figure 5:**
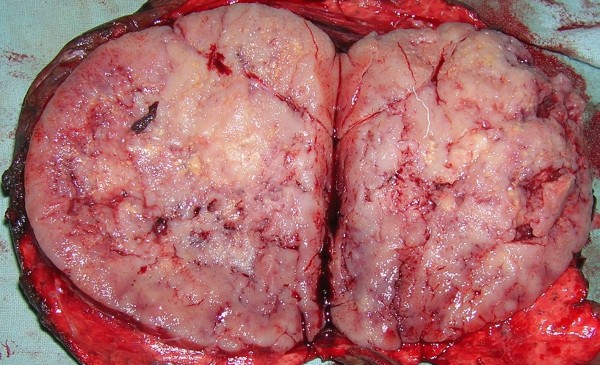
Bisected specimen with lingula.

Left postero-lateral thoracotomy through fifth intercostal space was done. Approximately 15×15 cm sized mass occupying entire left upper lobe (See Figure 4) not involving parietal pleura was noticed. The left upper lobe along with the tumor was reflected anteriorly and inferiorly and the left pulmonary artery was exposed by opening the posterior mediastinal pleura. An artery to the posterior segment, the apical-posterior arteries and an artery to the anterior segment were encountered and divided between ligatures. Distal dissection over the pulmonary artery led to the lingular arteries, which were also divided in ligatures. The anterior and posterior portions of the fissure were cut opened with the fine dissection and the parenchymal surface on the lower lobe was sutured with Polypropylene 3–0 suture utilizing continuous technique. The upper lobe bronchus was visualized clearly and closed with the 30 mm linear (TA) stapler. The distal branches of the superior pulmonary vein were ligated and transected and the lobe along with the tumor was removed (See Figure 5). Hilar lymph node sampling was performed, hemostasis achieved and the chest was closed routinely. The histopathology of the specimen was suggestive of well differentiated fetal adenocarcinoma (monophasic pulmonary blastoma) with clear margins without any evidence of tumor cells in lymph nodes. Post operative course was uneventful. Patient was given six cycles of Cisplatin based adjuvant chemotherapy and there was no evidence of recurrence or distant metastasis in two years of follow up.

## Discussion

Pulmonary tumors of embryonic origin are rare and among them, pulmonary blastomas are probably the most uncommon [3]. They form about 0.25 to 0.50% of all lung neoplasm, presumably arising from primitive pulmonary mesenchyme and histologically they resemble fetal lung [1]. They tend to occur peripherally and have a poor prognosis. First described by Barnard in 1952, they have been divided into three subgroups viz. classic pulmonary blastoma, well-differentiated fetal adenocarcinoma also called monophasic pulmonary blastoma and pleuropulmonary blastoma [1]. Classical pulmonary blastoma is the most common of these three subtypes. Some authors have classified the pulmonary blastoma into biphasic and Monophasic variety [4]. Biphasic pulmonary blastomas contain both neoplastic glandular tissue and either adult sarcomatous or embryonic mesenchymal tissue whereas Monophasic blastomas contain solely malignant glands of embryonic appearance [5]. A pulmonary blastoma should not be confused with the pleuropulmonary blastoma of childhood, which is analogous to Wilm’s tumor of the lung in which there is no recognizable neoplastic epithelium.

Approximately 25% to 40% of patients are asymptomatic at presentation, with incidental diagnosis by chest radiography. Common symptoms are cough, hemoptysis and chest pain while pleural effusion is seen unusually. Present case was having chest pain as a main complaint. The majority of pulmonary blastomas have been reported in adults, occurs mainly in young women [2] and is associated with smoking in 82% cases [1]. The monophasic type presents better prognosis compared to biphasic variety.

Radiography shows a well-demarcated peripheral lesion, well defined homogenous peripherally placed radiopacities with the lesions ranging from 2.5 to 25 cm [1,6]. On ultrasound examination, they shows heterogeneous appearance with solid and few cystic areas which indicate necrotic component. On computed tomography, heterogeneous appearance with typically enhancing whorls of solid tissue and no enhancing areas of necrosis are seen [6]. Metastases to same or opposite lung as well as involvement of mediastinum and adjacent chest wall can also be seen.

On gross pathology, the tumor is rounded, well circumscribed. In some, it may show lobulated appearance and multiple lesions. On immunohistochemistry expression of cytokeratin and vimentin is seen in the blastomatous component, the epithelial elements in blastomas react positively for keratin, carcinoembryonic antigen, epithelial membrane antigen, and milk fat globulin [7]. In the present case the tumor expressed cytokeratin, vimentin and keratin.

Surgery is the preferred treatment and is mandatory [8] whenever possible, often with adjuvant chemotherapy and/or radiotherapy [9]. Some authors suggest that the combination of surgery, adjuvant radiotherapy and chemotherapy based on cisplatin and etoposide should be considered [9]. Using an adjuvant protocol similar to the one used in the treatment of germ cell tumors (cisplatin, VP-16, uromitexan, ifosfamide and 64 Gy of mediastinal radiotherapy), a Swiss group has reported a 33-month survival in a stage III-A patient (pT3N2M0) [2]. In most health care facilities, radiotherapy is used to treat cases that do not respond to other forms of treatment [10]. Prognosis is worse if the tumour is larger than 5 cm at presentation [9]. Distant metastases are frequently seen in the liver, central nervous system, mediastinum, and bones [7]. This case was presented with 15×15 centimeters with no evidence of distant metastasis.

Overall, survival is 25% at 1 year and 16% at 5 years. Factors that indicate a poor prognosis are tumour recurrence, metastases at initial presentation, tumor greater than 5 cm, and lymph node metastases [1]. The prognosis is worse with the biphasic than monophasic type blastoma because of the high incidence of metastasis in the former [1,5]. No therapeutic guidelines exist. If metastases are present, survival is unlikely despite therapy.

## Conclusions

Monophasic pulmonary blastomas are rare neoplasms with good overall prognosis. Surgical excision is the treatment of choice and produce better prognosis with adjuvant chemotherapy.

## Consent

Written informed consent was obtained from the patients for publication of this Case report and accompanying images. A copy of the written consent is available for review by the Editor-in-Chief of this journal.

## Abbreviations

CT scan: Computed tomogram.

## Competing interests

The authors declare that they have no competing interests.

## Authors’ contributions

JHM drafted the manuscript, SBP analyzed and interpreted the patient data, HM performed the performed the surgery and was a major contributor in writing the manuscript, AFP conceptualize the manuscript and PNM involved in the crucial revisions of manuscript. All authors read and approved the final manuscript.
